# Left Threatened by Right: Political Intergroup Bias in the Contemporary Italian Context

**DOI:** 10.3389/fpsyg.2019.00026

**Published:** 2019-01-24

**Authors:** Michael Schepisi, Giuseppina Porciello, Ilaria Bufalari, Salvatore Maria Aglioti, Maria Serena Panasiti

**Affiliations:** ^1^Department of Psychology, Sapienza University of Rome, Rome, Italy; ^2^IRCCS, Santa Lucia Foundation, Rome, Italy; ^3^Department of Psychology of Developmental Processes and Socialization, Sapienza University of Rome, Rome, Italy

**Keywords:** political intergroup bias, ideological conflict hypothesis, personalized politics, perceived threat, entitativity, agentivity

## Abstract

Using different evaluation targets (i.e., politicians’ pictures, ideological words, items referring to features attributed to political ingroup/outgroup) we characterized the intergroup bias among political groups in the Italian context (Study 1-2-3) and tested a model that may account for the bias itself (Study 3). For all evaluation targets, left-wing participants - compared to right-wing participants – showed a greater intergroup bias, expressing more negative emotions toward the outgroup. The process was influenced by a greater perceived threat of the outgroup. Conversely, right-wing participants expressed the bias only when presented with ideological words. Our results provide a detailed description of how intergroup bias in Italy is differently expressed by the two ideological groups depending on the targets used to represent the political counterpart. Moreover, the results show that the stronger bias expressed by left-wing participants is driven by perceived threat of the outgroup.

## Introduction

Humans are social animals that form groups not only to survive in the world, but also to increase their sense of belonging, security (Correll and Park, 2005) and identity (Tajfel and Turner, 1979). Social categorization is the process by which people restrict their perception of social objects (Hogg and Abrams, 1988) and separate what is similar or dissimilar to themselves by coding others as ingroup vs. outgroup (Tajfel, 1969). Although it typically occurs in an automatic way (Brewer, 1988; Fiske and Neuberg, 1990), social categorization may be influenced by several variables such as the perceived warmth and competence of others (Ponsi et al., 2016), emotional reactivity (Ponsi et al., 2017b), self-uncertainty (Wagoner and Hogg, 2016), physical (Peck et al., 2013; Porciello et al., 2014) and personological similarity (Liuzza et al., 2011, 2013; Porciello et al., 2016), social interactions (Bufalari et al., 2014; Sacheli et al., 2015), and affect (Isen et al., 1992; Miller et al., 2010). These variables can in turn affect ethical, economic and social decisions (De Dreu et al., 2014; Shalvi and De Dreu, 2014; Panasiti et al., 2016; Ponsi et al., 2017a; Azevedo et al., 2018).

It has been found that a seemingly inevitable consequence of being part of a group (natural or created artificially for experimental purposes) is the so-called intergroup bias (Tajfel, 1969), which leads one to favor the ingroup and derogate the outgroup through positive/negative evaluations, emotions and behaviors. While the study of positive bias toward the ingroup has received much more attention than the derogation of the outgroup, it has been reported that the latter mechanism is very important for characterizing the intergroup bias (Mummendey et al., 1992; Brewer, 1999; Mummendey and Wenzel, 1999; Brown, 2000; Hodson et al., 2003; Hogg, 2003; Aquino and Becker, 2005; Park and Judd, 2005). Studies suggest that the predominance of one aspect on the other depends on the salience of specific needs and motivations (Levin and Sidanius, 1999). Outgroup derogation, often expressed by social and physical distance, negative emotions, intolerance, low cooperativeness and low pro-social behaviors (Haidt et al., 2003; Skitka et al., 2005; Mullen and Skitka, 2006; Morgan et al., 2010), seems to play a fundamental role in the dynamics of the so-called morality-based groups. In these groups -such as those surrounding the issue of abortion (Pro-life vs. Pro-choice)- the intergroup bias is the result of a differentiation from the “other” that is based on moral principles (Skitka et al., 2005; Krebs, 2008; Gray and Wegner, 2009; Horberg et al., 2009; Parker and Janoff-Bulman, 2013). Here, the intergroup bias is predominantly expressed by outgroup derogation, which can be displayed not only by directly harming others, but also by refraining from helping them (Weisel and Böhm, 2015). What seems to drive outgroup derogation for morality-based groups is the perceived threat of the outgroup (Parker and Janoff-Bulman, 2013). Importantly for the present research, in morality-based groups based on political affiliation, intergroup bias seems to entail a clear outgroup derogation (Janoff-Bulman, 2009) that can be greater than the one driven by race (Iyengar and Westwood, 2015). It is worth noticing that this pattern of results mainly derives from the study of political groups in contemporary Western context which is dominated by a natural opposition between two major ideologies: Conservatism and Liberalism (Heit and Nicholson, 2010). These two ideologies differ not only in the personality traits their adherents display (e.g., Liberals more open to experience and Conservatives more conscientious; Carney et al., 2008), but also in basic and higher order cognitive mechanisms, such as motivations (Jost et al., 2004, 2007, 2009; Thórisdóttir and Jost, 2011), emotional processing (Oxley et al., 2008), attentional orienting (Carraro et al., 2015), conflict monitoring (Amodio et al., 2007), and behaviors (e.g., consumption behavior; Fernandes and Mandel, 2014; Shi et al., 2017).

Importantly, the two groups also differ in how they shape their morality: Conservatives tend more toward ingroup loyalty, while Liberals seem more heavily reliant on individual motives related to harm/care (Graham et al., 2009). System Justification Theory suggests that these cognitive, personality and moral differences combine to explain why Conservatives show more prejudice compared to Liberals, who, conversely, are thought to be more open minded, favorable to diversity (Chirumbolo et al., 2004; De Zavala et al., 2010; Thórisdóttir and Jost, 2011) and sympathetic toward outgroup minorities (Robinson et al., 1995; Farwell and Weiner, 2000). Thus, while these studies hint at the existence of a difference in the level of prejudice shown by the two political groups (Jost et al., 2004; Jost, 2017), the so called Ideological Conflict Hypothesis suggests that Liberals and Conservatives are equally prejudiced since they both tend to favor those who share their own opinions and values, and derogate those who are in contrast with such values (Chambers et al., 2013; Brandt et al., 2014; Crawford, 2014). In this view, prejudice is one of the possible strategies a group can use to defend its own worldview when it is threatened (Brandt et al., 2014). Results from three independent laboratories testing this hypothesis showed that when facing a political counterpart openly opposed to their values – and thus perceived as threatening – both Liberals and Conservatives appear to be biased (i.e., they show equal dislike, political intolerance and willingness to discriminate; Brandt et al., 2014).

It should be noted that, differently from countries in which the vast majority of the studies reported above was conducted (mainly United States and United Kingdom), Italy has a multi-party political system, in which tracing a net divide between Conservative and Liberal political parties is more difficult. Nonetheless, in the last 25 years – with the disappearance of the dominant catch-all party “Democrazia Cristiana,” in which elements of both conservative and liberal ideologies were present (Paolucci, 2008) – two big coalitions emerged with a more conservative ideology in the center-right coalition and more liberal in the center-left. We are aware that a perfect overlap of Italian left and right-wing with Liberalism and Conservatism respectively might still be inaccurate. Thus, we believe that using the left- vs. right-wing dichotomy is more suitable for the Italian context and will refer to this in the following.

In the light of this multi-party political system, when collecting their political orientation we asked participants to self-define as right or left-wing. In doing so we asked them to answer by referring to their ideology and not the party they were voting for. This selection procedure was adopted also because some political parties in Italy (such as “Movimento 5 Stelle”) declare not to follow any of the two predominant ideologies and thus selecting participants according to the parties they were voting for could have been misleading.

To characterize the intergroup bias among political groups in the contemporary Italian context (from September 2015 to May 2016, i.e., when a left-wing government was in power), we presented to right-wing and left-wing Italian voters pictures of left and right-wing Italian politicians (Study 1), words related to left and right-wing ideologies (Study 2), and items referring to left and right-wing people (Study 3). We reasoned that varying the type of stimulus (pictures of politicians, ideological words, and items referring to the two political groups) could affect different processes and thus change the expression of the bias.

Indeed, it has been observed that - through mechanisms such as perceived voter-leader similarity (Liuzza et al., 2011, 2013; Cazzato et al., 2015) and attribution of authority and power (Porciello et al., 2016) – right-wing people tend to be more influenced than the left-wing ones by the presentation of their political leaders. Therefore, we expected that (Hypothesis 1) the use of pictures of politicians (Study 1) would lead to a higher intergroup bias (especially ingroup favoritism) in right-wing participants. In contrast, since ideological words (Study 2) might convey directly political ingroup–outgroup distinction without the mediation of the aforementioned processes, we expected that (Hypothesis 2) the expression of the intergroup bias would follow the predictions of the ideological conflict hypothesis (Brandt et al., 2014), with the same level of prejudice for both groups.

Finally, by presenting items related to participants’ political ingroup and outgroup (Study 3), we expected that the lack of personalization and authority cues would lead to confirm the results of Study 2 and extend the characterization of the bias not only at the emotional but also at the cognitive and behavioral level (Hypothesis 3). Moreover, to test what are the possible causes of the process that lead to the political intergroup bias, we measured certain factors that have been observed to play a role in the expression of intergroup bias either in natural and minimal groups (Gaertner and Schopler, 1998; Rubini et al., 2007; Effron and Knowles, 2015). Specifically, by relying on studies (those supporting the ideological conflict hypothesis and those on morality-based groups) that enlighten the role of perceived threat of the outgroup in the emerging of the bias (Janoff-Bulman, 2009; Parker and Janoff-Bulman, 2013; Wetherell et al., 2013; Brandt et al., 2014), we measured whether left and right-wing participants would exhibit a different level of perceived threat toward the outgroup and whether this could intervene in the expression of the bias. We expected that (Hypothesis 3.1) the more the outgroup was perceived as threatening the higher was the intergroup bias, expressed in particular in the form of outgroup derogation (Dovidio and Gaertner, 2010). In this vein, we also investigated whether entitativity (the extent to which a group is perceived as a group; Campbell, 1958) and agentivity (the extent to which a group is perceived as able to act as a group to achieve its goals; Abelson et al., 1998) could play a role and we expected that (Hypothesis 3.2) perceiving an outgroup as entitative and agentive could trigger a higher outgroup derogation. Conversely, perceiving the ingroup as entitative and agentive, and therefore able to defend its members, could enhance ingroup favoritism.

## Study 1

In Study 1 we asked left and right-wing Italian participants to (i) recognize, (ii) politically categorize and (iii) emotionally evaluate (by providing the valence of the elicited emotions) pictures of Italian politicians from both left and right-wing parties. Due to prior literature showing Conservatives to be more sensitive to authority – especially that of ingroup leaders (Liuzza et al., 2011; Porciello et al., 2016) – we expected right-wing participants to express more positive emotions than Liberals toward what they categorized as ingroup.

### Materials and Methods

#### Participants

Sixty six participants (33 females; age: *M* = 25.66 years, *SD* = ±7.15) were recruited by posting an invitation to complete an online survey regarding the categorization and evaluation of certain politicians. Sample size was determined following similar studies of our research group on social categorization (Ponsi et al., 2016). All the participants were Italian. Thirty one were right-wing and thirty five left-wing. The experimental procedures were approved by the independent Ethics Committee of the Santa Lucia Foundation in Rome (Scientific Institute for Research Hospitalization and Health Care) and were in accordance with the 1964 Declaration of Helsinki.

#### Materials and Procedure

The questionnaire was built and run through the online survey editor SurveyMonkey^TM^. Before starting the survey, participants were asked to read and accept the informed consent document by clicking with the mouse on a link which redirected them to the survey. Participants were presented with 58 pictures of Italian politicians in a randomized order, 29 left and 29 right-wing, with 17 males and 12 females in each group (for a complete list of the stimuli see Appendix). For each stimulus we asked participants (1) whether they recognized the politician (Recognition*: “Do you recognize the person in the picture?”).* Participants could either answer “*Yes*” or “*No.*” Those who responded affirmatively were asked to write the politician’s surname in order to ensure their recognition. Participants were then asked to (2) politically categorize the politician in the picture (Political Categorization*:* “*How would you politically categorize the person in the picture*?”). They replied using a four-step scale (i.e., “*Right-wing, Center-right, Center-left, Left-wing*”). Finally, we asked participants (3) to rate the emotions evoked by the politician (Valence*:* “*What kind of emotions does the person evoke in you*?”). Participants replied by using a 9-point Likert scale (i.e., “*1 = Extremely negative emotions; 9 = Extremely positive emotions”*). Participants who had not recognized the stimulus were allowed to skip the questions concerning valence and political categorization. This measure was our principal dependent variable both in this study and in Study 2. Although consisting of a single item, methodological research in psychology showed no substantial differences in terms of reliability between single-item and multiple-items measures (Wanous and Hudy, 2001; Dolbier et al., 2005). Importantly, although we checked for participants’ ability to assign politicians to the correct political group (accuracy of 95%; *SD* = ±0.18), we decided to use their response as a predictor rather than the actual political orientation of the politicians. The claims of this study are thus merely correlational, not causal, as both our dependent and independent variables are built on participants’ responses (i.e., they are not experimentally manipulated). After evaluating all politicians’ images, participants were asked to provide their demographic information (age, gender, nationality, occupation, education level, and country) and *Political Orientation* (henceforth named as *Group*) by indicating their ideology on a four-step scale (i.e., *Right-wing, Center-right, Center-left, Left-wing*).

#### Analysis and Results

**FIGURE 1:**
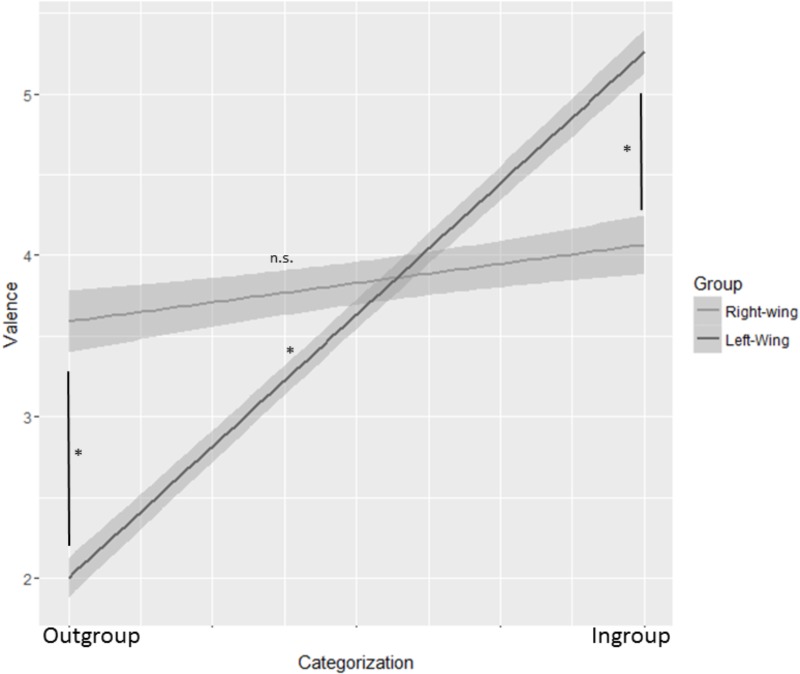
Mixed models interaction between Political Categorization and Group with politicians as stimuli. Political Categorization is shown on the *X*-axis. The slope was significantly different from 0 for left-wing participants (*p* < 0.001) but not for right-wing ones (*p* > 0.05). More specifically, left-wing participants expressed more positive emotions toward the stimuli categorized as ingroup and more negative emotions toward the outgroup stimuli compared to right-wing ones. The shaded bands represent 95% confidence intervals. ^∗^*p* < 0.001.

Data analysis was performed with R, a free software programming language and software environment for statistical computing (R Core Team, 2013). Trials in which participants did not recognize the politician were excluded from the analysis (Total valid trials = 62%; range: min = 13%, max = 100% of valid trials per subject). We then performed a multilevel mixed linear regression analysis (LMM or “mixed-effects models”; Pinheiro and Bates, 2000; Garson, 2013) through the package *lme4* Version 1.1–5 (Bates et al., 2014). Unlike traditional statistical methods, LMM are suitable for (a) analyzing hierarchical data structures (i.e., in which not all levels of a categorical factor co-occur at all levels of another categorical factor); (b) analyzing the whole data set (not just the mean observations for each subject and condition) to better evaluate the data variations that variance-style analyses (ANOVA) often leave out; (c) accounting for the non-independence of observations with correlated error; (d) separately treating the effects caused by the experimental manipulation (fixed effects) and those that were not (random effects) (Pinheiro and Bates, 2000). We used Valence as the dependent measure of our model. The fixed effects were the Political Categorization of the stimulus, the Group and their respective interactions. Political categorization of the stimulus was recoded as follows: if the categorization of the stimulus made by the participant matched his/her Group (i.e., *left* vs. *right-wing*), that stimulus was considered as *ingroup* (e.g., a stimulus categorized as left-wing by a left-wing participant was considered as ingroup). If the categorization did not match, that stimulus was considered as *outgroup* (e.g., a stimulus categorized as right-wing by a left-wing participant was considered as outgroup). We considered the random intercept over participants and the random slope of Political categorization over participants as random factors. Statistical significance of fixed effects was determined using type III Wald *F* tests with Kenward–Roger degrees of freedom (Kenward and Roger, 1997) and the Anova function from R’s *car* package. *Post hoc* pairwise comparisons (FDR corrected) were performed using least squares contrasts (lsc), as employed in R’s *lsmeans* package. The analysis revealed a significant Group × Political Categorization interaction *F*(1,70.025) = 48.31, *p* < 0.001. *Post hoc* analysis showed the slope of left-wing participants to be significantly different from zero (*b* = 3.11, *SE* = 0.26, df = 68.75, t.ratio = −11.94, *p* < 0.001), while that of right-wing participants was not (*b* = 0.41, *SE* = 0.28, df = 71.09, t.ratio = −1.43, *p* = 0.16). This indicates that, unlike left-wing, right-wing participants expressed no difference in emotional valence when evaluating ingroup or outgroup stimuli (see Figure 1). Furthermore, left-wing participants expressed more positive emotions toward ingroup stimuli (*M* = 5.17; *SE* = 0.23) than right-wing participants [*M* = 4.06; *SE* = 0.25; Mean difference = −1.1, *SE* = 0.35; *t*(65.98) = −3.177, *p* = 0.01, *r* = 0.36]. Left-wing participants also made more negative evaluations of the stimuli they had categorized as right-wing (*M* = 2.08; *SE* = 0.18) than right-wing participants did with stimuli they had categorized as left-wing [*M* = 3.65; *SE* = 0.20; Mean difference = 1.58, *SE* = 0.27, *t*(69.59) = 5.83, *p* < 0.001, *r* = 0.57].

### Discussion

Despite our expectations for a higher intergroup bias among right-wing participants, results from this first study show that only left-wing ones express the bias. Specifically, we found that, while right-wing participants’ emotions toward the stimuli are not related to the political categorization they make, left-wing ones express significantly different emotions according to the way they categorize the stimuli. When facing a stimulus that they recognize as belonging to their political ingroup or outgroup, left-wing participants tend to express more positive or negative emotions respectively when compared to right-wing ones. It should be noted, however, that this study was conducted while a left-wing – and not right – government was in power. For this reason, right-wing leaders’ authority might have been less strong and effective in leading right-wing participants’ bias, in line with what was found by Porciello et al. (2016).

## Study 2

Study 2 was identical to Study 1 except for the fact that words were used as stimuli instead of politicians’ pictures. The words were chosen to represent either ingroup or outgroup by conveying a right or left-wing ideology. Differently from politicians’ pictures, words refer to ideologies without involving the process of personalization or the different levels of sensitivity to authority. We thus expected that the ideological contrast contained in the words would lead right and left-wing participants to a similar expression of intergroup bias, as predicted by the ideological conflict hypothesis (Brandt et al., 2014). In particular, we expected both groups to express more positive emotions for the words that they categorized as ingroup and more negative emotions for the words they categorized as outgroup stimuli.

### Materials and Methods

#### Participants

Eighty two participants (52 left-wings and 30 right-wings) were recruited by posting an invitation to complete an online survey regarding the categorization and evaluation of political words. To have a better balance in the size of the two groups we selected through a random procedure a subsample of left-wing participants. Specifically, we generated a list of numbers ranging from 1 to 52 and randomly assigned them to our left-wing participants. Participants having a number comprised between 1 and 30 were then selected. Therefore, the final sample consisted of 30 left-wing and 30 right-wing participants (33 females; age *M* = 28.96, *SD* = ±9.67), in keeping with Study 1 and previous studies of this research group on social categorization (Ponsi et al., 2016). All the participants were Italian. The experimental procedures were approved by the independent Ethics Committee of the Santa Lucia Foundation in Rome (Scientific Institute for Research Hospitalization and Health Care) and were in accordance with the 1964 Declaration of Helsinki.

#### Materials and Procedure

The questionnaire was built and run through the online survey editor SurveyMonkey^TM^. Before starting the survey, participants were asked to read and accept the informed consent document by clicking with the mouse on a link which redirected them to the survey. Forty six words were selected from specific political scales such as the Right-Wing Authoritarianism – RWA (Altemeyer, 1998) – and the Social Dominance Orientation – SDO – (Tilly et al., 2001). The words were selected and categorized on the basis of the ideological policy expressed – half representing a right-wing, half a left-wing ideology – and tested by the questionnaire items (see Appendix for a complete list of the stimuli). The words did not differ for lexical frequency [*M*_right_= 1.45, *SD*_right_= ±0.80; *M*_left_= 1.51, *SD*_left_= ±0.80; *t*(44) = −0.284, *p* = 0.81] or character length [*M*_right_= 1.00, *SD*_right_= ±0.09; *M*_left_= 0.98, *SD*_left_= ±0.14; *t*(44) = 0.520, *p* = 0.60]. Both the order in which the words were presented and the questions appeared within the survey were randomized.

Similarly to Study 1, for each stimulus we asked participants to (1) politically categorize the ideological word (Political Categorization: “*Which ideology do you think represents the word above?*”). Participants replied by using a four-option scale (i.e., “*Right-wing, Left-wing, Both of them, Neither of them”*). Participants were then asked (2) to rate the emotions evoked by the word (Valence: “*What kind of emotions does the word evoke in you*?”). Here replies were made by using a 9-point Likert scale (i.e., “*1 = Extremely negative emotions; 9 = Extremely positive emotions”*). As in Study 1, this measure was employed as dependent variable. Again, although we checked that participants were able to correctly assign the words to the corresponding political group (accuracy of 95%; *SD* = ±0.18), we decided to use their responses as a predictor, rather than the actual political orientation of the words. Thus, the claims also of this study are merely correlational, not causal. Finally, we asked the same demographic information and question on *Political Orientation* (henceforth called *Group*) as in Study 1.

#### Analysis and Results

**FIGURE 2:**
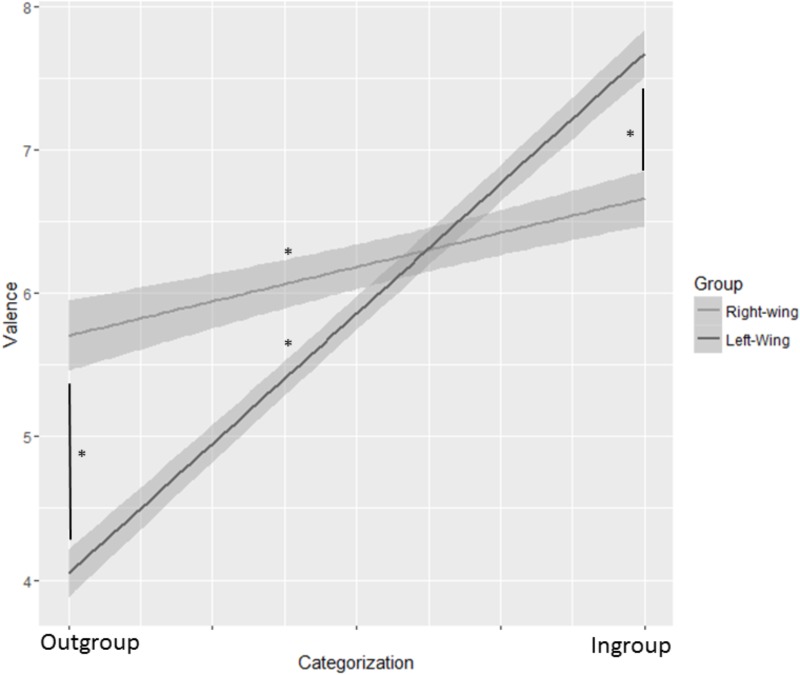
Mixed models interaction between Political Categorization and Group with ideological words as stimuli. Political Categorization is shown on the *X*-axis. The slopes for both left and right-wing participants differed from 0 (all *p*s < 0.003), but left-wing participants showed significantly more negative emotions toward the outgroup and more positive emotions toward the ingroup compared to right-wing ones. The shaded bands represent 95% confidence intervals. ^∗^*p* < 0.003.

Trials in which participants could not assign the word to a specific political category (i.e., they indicated that the word was representing both or neither of the two ideologies) were excluded from the analysis (Valid trials = 57.1%; range: min = 6%, max = 95% of valid trials per subject). We performed the same linear mixed model as in Study 1, revealing a significant Group x Political Categorization interaction [*F*(1,58.412) = 34.34, *p* < 0.001]. *Post hoc* analysis showed that the slopes of both left (*b* = 3.56, *SE* = 0.31, df = 55.11, t.ratio = −11.24, *p* < 0.001) and right-wing participants (*b* = 0.86, *SE* = 0.33, df = 61.64, t.ratio = −2.59, *p* < 0.001] were different from zero, indicating that with this type of stimuli (i.e., ideological words) both groups showed an intergroup bias. Once again, however, this bias was stronger for left than right-wing participants. Specifically, left-wing participants expressed more positive emotions toward the stimuli they categorized as belonging to their ingroup (*M* = 7.59; *SE* = 0.24) than right-wing participants (*M* = 6.64, *SE* = 0.18); (*b* = −0.95, *SE* = 0.26, df = 56.51, t.ratio = −3.61, *p* = 0.003; *r* = 0.43) (see Figure 2). Furthermore, left-wing participants made less positive evaluations of the stimuli they had categorized as belonging to the outgroup (*M* = 4.02; *SE* = 0.22) than right-wing participants did (*M* = 5.77; *SE* = 0.33); (*b* = 1.74, *SE* = 0.33, df = 57.35, t.ratio = 5.20, *p* < 0.001, *r* = 0.56).

### Discussion

Changing the nature of the stimuli (i.e., using ideological words instead of politicians’ pictures) did not significantly change the pattern of results found in Study 1, but it did highlight a new, interesting, element that was in line with our hypothesis. While stronger in left-wing, intergroup bias was also found among right-wing participants. It seems that presenting ideological words makes the ideological conflict between the two groups more salient, leading them to express intergroup bias. However, again this conflict seems to affect left more than right-wing participants.

## Study 3

Study 3 was performed with the aim of (i) replicating the findings of Studies 1–2 and (ii) revealing potential influencal factors that could play a role in the different expression of the bias between left and right-wing participants. Participants were presented with items that referred to people representing their political ingroup/outgroup (namely, left and right-wing people). Because of the lack of personalization and authority cue, we expected to replicate the findings of Study 2 regarding the emotional bias and to find additional evidence for cognitive and behavioral bias in both left and right-wing participants. Moreover, literature on morality-based groups and research on ideological conflict hypothesis have underlined how the interaction between two opposing groups is often characterized by the presence of a perceived threat of the outgroup (Parker and Janoff-Bulman, 2013; Brandt et al., 2014). In addition, when perceiving a group as such (entitativity) that group could become more threatening, especially if attributed with the ability to act (agentivity), as its capacity for harm increases. This perception often leads to intergroup bias expressed by a more positive attitude toward the ingroup and a more negative attitude toward the outgroup (Dovidio and Gaertner, 2010). We thus investigated whether these variables (i.e., entitativity, agentivity and perceived threat) could play a role in the process that leads to the emergence of the bias.

### Materials and Methods

#### Participants

Seventy one participants (41 left-wing and 30 right-wing) were recruited by posting an invitation to complete an online survey regarding political opinions and evaluations. The same random selection procedure used in Study 2 was employed here to select a subsample of 30 left-wing participants. The final sample consisted of 30 left-wing and 30 right-wing participants (41 females, age *M* = 26.80; *SD* = ±5.44). All participants were Italian. The experimental procedures were approved by the independent Ethics Committee of the Santa Lucia Foundation in Rome (Scientific Institute for Research Hospitalization and Health Care) and were in accordance with the 1964 Declaration of Helsinki.

#### Materials and Procedure

The questionnaire was built and run through the online survey editor SurveyMonkey^TM^. Before starting the survey, participants were asked to read and accept the informed consent document by clicking with the mouse on a link which redirected them to the survey. We asked participants the same demographic information and the same question on *Political Orientation* (henceforth called as *Group*). Depending on the answer to this question, they were directed to a specific survey, one for left-wing and one for right-wing participants. The only difference between the two was the measure of perceived threat, which was adapted for their respective outgroup. Then, participants were asked to answer questions on political opinions and evaluations that measured entitativity, agentivity, and perceived threat as related to their political ingroup and/or outgroup (see Appendix for a complete list of the items). We also measured their intergroup bias on three domains (see Measures paragraph for a more detailed description). Items for each measure were presented in a randomized order.

#### Measures

*Entitativity* of both the ingroup and the outgroup was assessed with 8 items (adapted by Spencer-Rodgers et al., 2007) asking participants to express their agreement or disagreement on a 7-point scale (“1 = Strongly disagree; 7 = Strongly agree”). *“How much group unity do you think left/right-wing people feel?”* and *“How much do left/right-wing people interact with one another?”* are two examples of the items.

*Agentivity* is a specific aspect of entitativity related to the ability to act as a group. It was assessed for both the ingroup and outgroup with four items (adapted by Spencer-Rodgers et al., 2007), in which participants were asked to express their agreement or disagreement with the sentences on a 7-point scale (“1 = Strongly disagree; 7 = Strongly agree”). *“To what extent are left/right-wing people able to act collectively?”* and *“To what extent are left/right-wing people able to achieve their goals?”* are two examples of the items.

*Perceived threat* toward the outgroup was assessed with five items adapted from Schmid and Muldoon (2015). In order to adjust this measure to the others, and to have a more sensible tool, we decided to use a 7-point scale instead of the original 5-point scale to assess participants’ agreement or disagreement with the items (“1 = Strongly disagree; 7 = Strongly agree”). *“I feel threatened if left/right-wing are in power in Italy”* and *“When I see left/right-wing symbols I feel as though my identity is under threat”* are two examples of the items.

We also measured the *Intergroup Bias* on three different dimensions: emotional, cognitive and behavioral. Again, each measure was taken for the political ingroup and outgroup (i.e., left/right-wing).

*Emotional Intergroup bias* was assessed with the General Evaluation Scale, a feeling thermometer taken from Wright et al. (1997). This measure is composed of 6 bipolar noun pairs separated by a 7-point scale. Participants were asked to express their feelings toward an ingroup (*Emotion Ingroup*) or outgroup (*Emotion Outgroup*) person. Examples of these pairs were *warmth-coldness* and *negativity-positivity.*

*Cognitive Intergroup bias* was assessed with 12 traits taken from Chambers and Melnyk (2006). Participants were asked to indicate how much they thought each trait represented a person of the ingroup (*Cognition Ingroup*) or outgroup (*Cognition Outgroup*) on a 10-point scale (“*1 = Not at all; 10 = Very much”*). Examples of these traits were *Intelligent, Honest, Immoral* and *Radical*.

*Behavioral Intergroup bias* was assessed with two items. One of these items – again taken from Wright and colleagues (Wright et al., 1997) – asked the participants to decide how they would distribute 500 euro between a left/right-wing person. As this measure, in our opinion, only took the ingroup favoritism aspect of the bias into account, we decided to create a second item which could address the behavioral outgroup derogation as well. Participants were thus asked to decide how they would take 500 euro from a left/right-wing person. Participants were reminded for both measures that the total amount given or taken away had to be 500 euro. Finally, *Political Orientation (Group)* was measured as in Study 1 and Study 2.

#### Analysis and Results

The analysis was conducted using IBM SPSS Statistics 22 and STATISTICA 7 softwares. Since we did not have multiple trials per condition per participant in this experiment, but single measurements per construct per participant, there was no need to use mixed models analysis. All our dependent measures were normally distributed except for the measure of Emotional Intergroup bias (ingroup and outgroup) [Kolmogorov–Smirnov *d*(60) = 0.145, *p* < 0.20], which had four outliers. Outlier participants were defined as those presenting mean values above ± 2.5 standard deviations from the grand mean of all participants in each single condition, and recoded by using the mean value of the respective condition ± 2.5 standard deviations as indicated by Field (2009). Separate 2 × 2 analysis of variance (ANOVA) with Target (ingroup vs. outgroup) as within-subject factor and Group (left vs. right-wing) as between-subject factor were performed on Entitativity, Agentivity, Emotional Intergroup Bias, Cognitive Intergroup Bias and Behavioral Intergroup Bias. *Post hoc* comparisons were conducted, when necessary, by means of the Duncan test. Finally, independent sample *T*-test was performed on Perceived Threat of the outgroup as dependent variable and Group (left vs. right-wing) as between-subject factor.

##### Entitativity results

**FIGURE 3:**
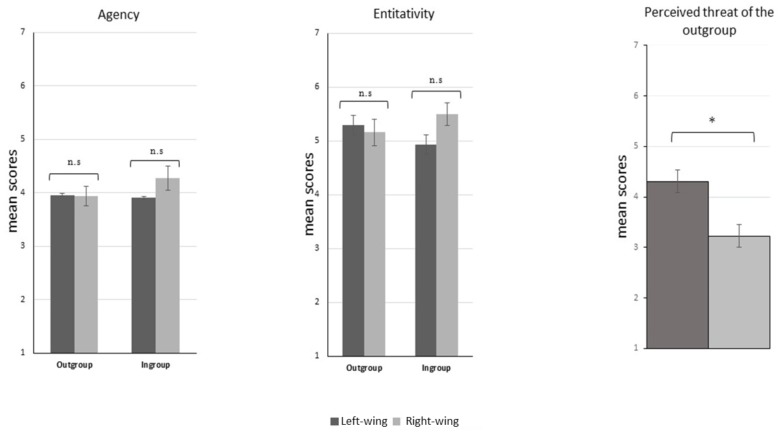
Agentivity, Entitativity, Perceived threat. Comparisons of the mean scores between left and right-wing participants on ingroup/outgroup Agentivity, ingroup/outgroup Entitativity and Perceived threat of the outgroup. The only significant comparison between left and right-wing participants was on Perceived threat of the outgroup, with higher scores for former compared to the latter. ^∗^*p* < 0.001.

This scale had a high reliability both for the ingroup (Cronbach’s α = 0.89) and the outgroup (Cronbach’s α = 0.86). The ANOVA on Entitativity revealed no main effects of Target [*F*(1,58) = 0.003, *p* = 0.95, $\eta_{p}^{2}$ = 0.00] or Group [*F*(1,58) = 0.809, *p* = 0.37, $\eta_{p}^{2}$ = 0.01] and no Target × Group interaction [*F*(1,58) = 2.141, *p* = 0.14, $\eta_{p}^{2}$ = 0.03], indicating that the two groups did not differ in their perception of ingroup vs. outgroup entitativity (see Figure 3).

##### Agentivity results

This scale had a high reliability for the ingroup (Cronbach’s α = 0.83) and a medium reliability for the outgroup (Cronbach’s α = 0.71). The ANOVA on Agentivity revealed no main effects of Target [*F*(1,58) = 0.609, *p* = 0.43, $\eta_{p}^{2}$ = 0.01] or Group [*F*(1,58) = 0.738, *p* = 0.39, $\eta_{p}^{2}$ = 0.01] and no Target × Group interaction [*F*(1,58) = 1.147, *p* = 0.28, $\eta_{p}^{2}$ = 0.01], indicating that the two groups did not differ in their perception of ingroup vs. outgroup agentivity (see Figure 3).

##### Perceived threat results

This scale had a good reliability (Cronbach’s α = 0.74). The independent sample *t*-test showed left-wing participants to be more threatened by the outgroup (*M* = 4.30, *SE* = 0.22; *SD* = ±1.23) compared to right-wing participants (*M* = 3.22, *SE* = 0.21; *SD* = ±1.17) [*t*(1,58) = −3.471, *p* < 0.001, *r* = 0.41] (see Figure 3).

##### Emotional intergroup bias results

**FIGURE 4:**
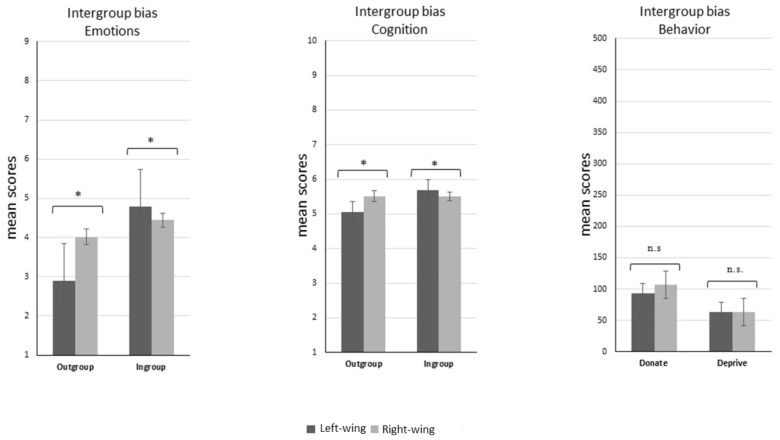
Intergroup bias on Emotions, Cognition and Behavior. Comparisons of the mean scores between left and right-wing participants on the three measures of intergroup bias: emotions, cognition and behavior. A significant difference between the two groups was found in emotions and cognitions (all *p*s < 0.03), but not in behavior (*p* = 0.79). This last measure was indexed in Donation using the formula ingroup donation – outgroup donation and in Deprivation using the formula ingroup deprivation – outgroup deprivation. ^∗^*p* < 0.03.

This scale had a high reliability for the ingroup (Cronbach’s α = 0.93) and the outgroup (Cronbach’s α = 0.95). The ANOVA on Emotional Intergroup Bias revealed a main effect of Target [*F*(1,58) = 28.700, *p* < 0.001, $\eta_{p}^{2}$ = 0.33] with the emotions toward the outgroup as more negative than those toward the ingroup and a main effect of Group [*F*(1,58) = 7.287, *p* < 0.05, $\eta_{p}^{2}$ = 0.11] with left-wing participants reporting more intense emotions compared to right-wing participants. The interaction was also significant [*F*(1,58) = 13.443, *p* < 0.001, $\eta_{p}^{2}$ = 0.18], revealing, in particular, that left-wing participants expressed significantly less positive (and so more negative) emotions toward the outgroup than right-wing participants did (*p* < 0.001). *Post hoc* analysis on emotions toward the ingroup revealed no differences between the two groups (*p* = 0.20) (see Figure 4).

##### Cognitive intergroup bias results

This scale had a medium-low reliability for the ingroup (Cronbach’s α = 0.67) and the outgroup (Cronbach’s α = 0.64). The ANOVA on Cognitive Intergroup Bias revealed a main effect of Target [*F*(1,58) = 8.011, *p* < 0.001, $\eta_{p}^{2}$ = 0.12] with more negative evaluation of the outgroup compared to the ingroup, but no main effect of Group [*F*(1,58) = 0.663, *p* = 0.41, $\eta_{p}^{2}$ = 0.01]. The Target × Group interaction showed to be significant [*F*(1,58) = 8.466, *p* < 0.001, $\eta_{p}^{2}$ = 0.12], again revealing that left-wing participants made significantly less positive evaluations of the outgroup (*M* = 5.05, *SD* = ±0.79) with respect to the ingroup (*M* = 5.67, *SD* = ±0.88) (*p* = 0.03) than right-wing participants did (outgroup: *M* = 5.51, *SD* = ±0.84; ingroup: *M* = 5.50, *SD* = ±0.67) (see Figure 4).

##### Behavioral intergroup bias results

The ANOVA on Behavioral Intergroup Bias revealed no main effects of Target [*F*(1,58) = 2.077, *p* = 0.15, $\eta_{p}^{2}$ = 0.03] or Group [*F*(1,58) = 0.031, *p* = 0.86, $\eta_{p}^{2}$ = 0.00] or Target × Group interaction [*F*(1,58) = 0.06, *p* = 0.79, $\eta_{p}^{2}$ = 0.00], indicating that there was no difference among the two groups in the way they behave toward the ingroup or the outgroup (see Figure 4).

##### Correlations

Bivariate correlations were computed between the variables of interest. The accepted alpha level of the p-value was Bonferroni corrected for the maximum number of comparisons for each variable: 0.05/3 = 0.017.

Results indicated that, while there was an inverse relationship between Perceived Threat and Emotion outgroup [*r*(60) = −0.50, *p* < 0.001], this relationship was not significant with Cognition outgroup [*r*(60) = −0.06, *p* = 0.62], indicating that threat seems to be involved at an emotional level of information processing related to the outgroup, rather than at a cognitive level. Moreover, Emotional bias toward the outgroup had no other relationships, neither with Agentivity [*r*(60) = 0.19, *p* = 0.14) nor with Entitativity [*r*(60) = 0.16, *p* = 0.20].

Differently, Cognition outgroup had a positive relationship with Agentivity [*r*(60) = 0.36, *p* = 0.004] but not with Entitativity [*r*(60) = 0.29, *p* > 0.017].

**FIGURE 5:**
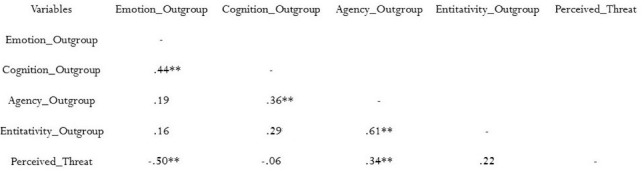
Correlations matrix among Emotion, Cognition, Agentivity, Entitativity, and Perceived Threat toward the outgroup. *r* Pearson’s coefficients are reported. *N* = 60; ^∗∗^*p* < 0.017, corrected for multiple comparisons. Two-tailed pairwise correlation.

In addition, we tested correlations between Perceived Threat, Agentivity and Entitativity. Results showed Perceived Threat to have a significant (and positive) relationship with Agentivity [*r*(60) = 0.34, *p* = 0.007], but not with Entitativity [*r*(60) = 0.22, *p* = 0.09], indicating that a group can be perceived as threatening not because of its “groupness,” but because of its potential to act (see Figure 5).

##### Mediation analysis

Since the two political groups differed in Perceived Threat of the outgroup in Emotional and Cognitive Intergroup Bias we used mediation analysis to assess whether the effect of Group on intergroup bias toward the outgroup (i.e., the dependent variable) was mediated by Perceived Threat. Moreover, although the two groups did not differ either in entitativity and agentivity, we run a mediation analysis also with Entitativty and Agentivity as mediators of the relationship Group and Emotional (and Cognitive) Intergroup bias. The product-of-coefficients strategy with bootstrapping was used to test strength and significance of the indirect effect (Preacher and Hayes, 2004; Preacher et al., 2007). In doing so we used the PROCESS macro implemented for IBM SPSS (Hayes, 2012).

##### Mediation model with emotional intergroup bias as dependent variable

**FIGURE 6:**
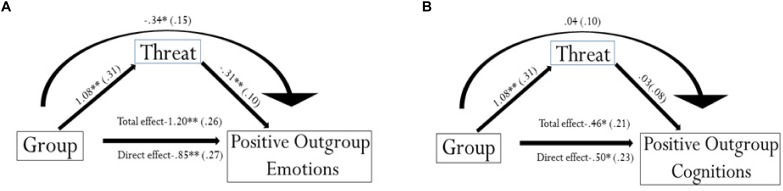
**(A)** Shows the Mediation model with Group, Perceived Threat and Emotions Outgroup. The predictor variable Group was coded 1 for right and 2 for left-wing participants. This means that an increase of 1 political orientation – namely, being left-wing – indicates a decrease in positive emotions toward the outgroup (and thus an increase in negative ones). The direct path remained significant after inserting Perceived Threat, but the effect size decreased from –1.20 to –0.85, *p* < 0.01. The indirect effect of X on Y mediated by M was significantly different from 0 (*B* = –0.34, Boot Se.1592, BootLLCI –0.7030, BootULCI –0.1056, *p* = 0.02). **(B)** Shows Mediation model with Group, Perceived Threat and Cognition Outgroup. The predictor variable Group was coded 1 for right and 2 for left-wing participants. Thus, an increase of 1 political orientation – namely, being left-wing – indicates a decrease in positive traits assigned to the outgroup (and an increase of negative ones). The path from the mediator to the dependent variable was not significant *p* > 0.05. The direct path was significant, but the indirect path was not (*B* = 0.0414, Boot SE.1097, BootLLCI –0.1831, BootULCI.2573). ^∗^*p* < 0.05; ^∗∗^*p* < 0.01.

We first determined that Group significantly predicted the hypothesized first mediator (Perceived Threat of the outgroup) [*b* = 1.08, *p* = 0.001, 95% Confidence Interval (CI)]. Then we tested that Emotion outgroup was predicted by using participants’ Group (left vs. right-wing). The regression was significant (*b* = −1.20, *p* < 0.001, 95% CI), and this relationship remained significant even after inserting Perceived Threat as mediator (*b* = −0.85, *p* = 0.002, 95% CI), but with a weaker effect, which indicates a partial mediation in the model. The 95% CI for the indirect path ranged from −0.7492 to −0.1120, indicating that the indirect effect was significantly different from zero at *p* < 0.05 (see Figure 6). This result suggests that Perceived Threat plays a crucial mediating role in the relationship between the two political Groups and the emotional aspect of the intergroup bias.

Conversely, the same model did not show any indirect effect of entitativity (Lower Confidence Interval = −0.1235; Upper Confidence Interval = 0.1516) or agentivity (Lower Confidence Interval = −0.1545; Upper Confidence Interval = 0.1571), indicating that these two factors did not play a role in modulating the emotional aspect of the intergroup bias depending on the political orientation of our participants.

##### Mediation model with cognitive intergroup bias as dependent variable

As above, Group predicted Perceived Threat (*b* = 1.08, *p* = 0.001, 95% CI). Cognition outgroup was regressed on Group and showed a significant relationship (*b* = −0.46, *p* = 0.03), even after inserting Perceived threat as mediator (*b* = −0.50, *p* = 0.03). The indirect path was not significant, as indicated by the 95% CI ranging from −0.1661 to 0.2792 with *p* > 0.05 (see Figure 6). Thus, Perceived Threat did not play any mediating role in this model. Similarly, as showed by their non-significant indirect effects, neither entitativity ((Lower Confidence Interval = −0.1290; Upper Confidence Interval = 0.1768) nor agentivity (Lower Confidence Interval = −0.1841; Upper Confidence Interval = 0.1750) played a role in this process. This suggests that, differently than for emotional bias, other variables not investigated here could account for the differences in cognitive intergroup bias between the two groups.

### Discussion

In this third study we tested whether the pattern of results found in the previous two studies could be altered by the presentation of items referring to participants’ political ingroup and outgroup as stimuli. We also explored the intergroup bias at an emotional, cognitive and behavioral level. Moreover, we explored whether variables such as entitativity and agentivity of the ingroup and outgroup, as well as threat perception of the outgroup, could influence the expression of the intergroup bias.

Left-wing participants expressed intergroup bias while right-wing ones did not, as in Study 1. The bias emerged not only at an emotional level, but also at the cognitive one, while no differences were found at a behavioral level. More specifically, while the two groups did not differ in their cognitive and emotional evaluations of the ingroup, left-wing participants expressed significantly more negative emotions toward – and worse cognitive evaluations of – the outgroup compared to right-wing participants. Mediation analysis showed that perceived threat of the outgroup influenced the effect in the emotional domain, but not in the cognitive one. This suggests that other latent factors could contribute to the explanation of this phenomenon. In contrast with our expectations, those factors were neither entitativity nor agentivity. Indeed, the ANOVA showed no differences between the two groups, either for the ingroup or outgroup.

## General Discussion

In three different studies we investigated the political intergroup bias showed by left and right-wing participants in the contemporary Italian context. As a main element of novelty we used different types of stimuli to represent the political target of evaluations with in mind the idea that changing nature of the political target could affect the expression of the intergroup bias. Classical studies indicate that Conservatives, because of their motivational and epistemic motives rely more often on stereotypes and express more prejudice toward other groups than Liberals (Chirumbolo et al., 2004; De Zavala et al., 2010). However, there is also evidence to suggest that both groups are equally capable of expressing prejudice toward groups that do not share their values or opinions, and that they perceive as a threat to their worldview (Chambers et al., 2013; Brandt et al., 2014). Motivated by the fact that both top–down and bottom–up mechanisms related to political perception can reciprocally affect each other (Castelli and Carraro, 2011; Liuzza et al., 2011; Alabastro et al., 2013; Porciello et al., 2016), we presented different types of stimuli as categorization targets (i.e., politicians’ pictures, ideological words and items referred to political ingroup/outgroup) in order to systematically investigate the bias. We thought that the use of politicians’ pictures was particularly important because of personalization (McAllister, 2007), i.e., the process by which electors come to rely more on personality-related variables of political leaders than on ideologies, policies and political programs (Katz and Mair, 1995; Caprara and Vecchione, 2016). Indeed, thanks to the increasing influence of TV and social media over the last two decades, the relationship between electors and politicians has been fundamentally shaped by the latter’s appearance. We hypothesized that the personalization process, together with one’s sensitivity toward authority, would drive the categorization of politicians’ pictures (Study 1), resulting in a higher bias for right-wing participants, while the other two stimuli -ideological words (Study 2) and written items referring to left and right-wing people (Study 3)- would induce a comparable bias in the two groups. In contrast with studies indicating that intergroup bias is stronger in Conservatives, our results showed left-wing participants to be more biased than right-wing ones, who did not express any bias in two of the three studies. It is worth noting that a prior study using surveys, focus groups and interviews also found more bias in left-wing Italian voters than in right-wing ones (Catellani, 2006). Yet our results could be explained by the plasticity of the personalization process, which relies on the real, moment-by-moment social status of ingroup/outgroup political leaders. This would be in keeping with our previous research showing that right-wing people, differently from left-wing ones, reduce their leader-voter perceived similarity and their tendency to follow the gaze of the leader according to his/her social status (Porciello et al., 2016). We speculate that right-wing participants, being more sensitive to authority (Altemeyer, 1998) and social hierarchies than left-wing ones (Tilly et al., 2001), may take their leaders’ current social status into account when evaluating them. The fact that there was a left-wing government during data collection may explain why, when politicians’ pictures were presented in Study 1, right-wing participants did not show ingroup favoritism.

In Study 2, where ideological words were employed, intergroup bias was present in both left and right-wing participants. This result could be explained by the fact that words do not initiate the personalization process, allowing ideological opposition and the subsequent emotional activation to prevail, and intergroup bias to occur in both groups. Interestingly, though, left-wing participants’ bias was actually greater than the one expressed by right-wing participants, showing either more ingroup favoritism and outgroup derogation. Study 3 provides clues on the factors that could have played a role in the different expression of the political intergroup bias in the two groups. We asked participants to answer items containing an emotional, cognitive and behavioral evaluation of the political ingroup/outgroup. Left-wing participants again showed a higher bias than right-wing ones at the emotional and cognitive levels, but no differences between the two groups were found in the behavioral domain. However, there are at least two problems with this measure: first, by referring to the intention to act in a certain manner rather than expressing an actual behavior, it might be influenced by experimenter demand effects. Second, as for every other aspect in the present study, we used a self-report measure, which suffers from limitations that have been deeply acknowledged in social psychology literature, such as participants’ social desirability and scarce introspection skills (Nisbett and Wilson, 1977). In addition, the two questions that we presented lacked specific contextual information (e.g., how much money they had before donating), which might have decreased the sensitivity of the measure and, thus, made it hard to justify such a deliberate and morally relevant decision.

Conversely, when asked to express an emotional evaluation and to assign positive or negative traits to the ingroup and outgroup, left-wing participants again showed a greater bias, particularly in the form of outgroup derogation. With this regard, in keeping with research on morality-based groups (Halevy et al., 2011; Parker and Janoff-Bulman, 2013; Weisel and Böhm, 2015) and on sacred values (Tetlock, 2003; Ginges et al., 2007), we found outgroup derogation as the predominant expression of the bias throughout the three studies. In fact, differences between political groups are based on moral values that are considered as “sacred,” that is transcendent from any trade-off or contamination with other values (Tetlock et al., 1996; Parker and Janoff-Bulman, 2013). When these moral values are perceived as conflicting or being violated people experience threat (Weisel and Böhm, 2015). In turn, threat leads to strong outgroup derogation, which in some cases assumes the form of moral outrage, namely a state characterized by particular negative emotions (e.g., anger and contempt) and behaviors (e.g., harsh punishment) (Tetlock, 2003). Relatedly, we found a mediating role of perceived threat of the outgroup at the emotional level of the bias. This result, which is in line with previous research (Brandt et al., 2014), supports the idea that when the ingroup is threatened the motivation to protect the group (as well as one’s self) leads to express especially negative emotions toward the source of the threat, namely the outgroup. We also tested whether other variables besides threat could have played a role in the regulation of this process, possibly by interacting with perceived threat. We focused on perceived entitativity (the extent to which a group is perceived as a group; Campbell, 1958) and agentivity (the extent to which a group is perceived as able to act as a group to achieve its goals; Abelson et al., 1998) (referred to ingroup and the outgroup), because these variables were observed to modulate the intergroup bias in minimal and natural groups (see e.g., Gaertner and Schopler, 1998; Rubini et al., 2007; Effron and Knowles, 2015). On the one hand, we hypothesized that the more an outgroup is perceived as an acting group, the more participants would show outgroup derogation. On the other, we hypothesized that perceiving the ingroup as entitative and agentive could have increase ingroup favoritism.

No group difference or indirect effects of these two variables in mediating the relationship between the political group and the emotional and cognitive bias were found, making any further speculation regarding their role in influencing the intergroup bias impossible. We expected a bigger sense of entitativity attributed to the right-wing also because of the historical divisions characterizing Left-wing parties in Italy. This somewhat surprising result might be explained by the political situation at the time of the data collection. In facts, after the 2014 political elections, both the big center-right coalition guided by “Popolo della Libertà” and the center-left one guided by “Partito Democratico” started fragmentizing into smaller parties, an event that could have undermined the sense of unity both in right and left-wing voters.

To sum up, our studies suggest that: (i) in the Italian context left-wing people express in general more intergroup bias than right-wing people do; (ii) left- and right wings, show equal prejudice when the oppositional nature is made salient (as suggested by the ideological conflict hypothesis or the studies on the sacred values, (Tetlock, 2003; Brandt et al., 2014); (iii) the left-wing people’s higher prejudice seems to be mediated by perceived threat of the outgroup (as shown in Study 3); (iv) the inability of politicians’ pictures and political items to induce the bias in right-wing participants may have been due to their sensitivity to the authority of ingroup leaders, who were not in charge at the time of the study.

Thus, by showing that political intergroup bias might depend less on being left or right-wing *per se* than on the wider political situation, the proposed model is useful in describing the political context in Italy, where the left-wing has historically been weak (Vampa, 2009). The last three decades, in particular, which have seen left-wing parties across Europe transform from “catch-all parties” to “cartel-parties” (i.e., parties that are controlled and managed by professional politicians as an instrument aimed specifically at winning the elections; Vampa, 2009) has driven the Italian left-wing to renounce a part of its political identity. An open crisis and loss of consensus has resulted (Vampa, 2009), perhaps lessening the threat of the Left as perceived by the Right. On the other hand, we might speculate that the loss of the original political identity by the Left could, in turn, affect its voters by undermining their political (and social) identity. In this view, intergroup bias, might be just one strategy to reaffirm one’s own identity (Brewer, 1999). Moreover, 2008 crisis has produced economic and social instability that has contributed to growing populism in Italy as well as all over the world. Most of these populistic political movements seem to have their roots in the right-wing ideology (see for instance “Golden Dawn” in Greece, “Lega Nord” in Italy and “Le Front National” in France). Thus, their spreading might have contributed to an increase of the perception of threat and, in turn, of outgroup derogation by left-wing people. Extreme examples of this are represented by the episodes reported by Italian Media of assaults by left-wing activists during right-wing manifestations (e.g., Frignani, 2016)^1^. All in all, our results seem to support the ideological conflict hypothesis (Brandt et al., 2014), which states that groups express prejudice toward each other because of perceived ideological dissimilarity and the perceived threat of the outgroup (Brandt et al., 2014; Crawford and Pilanski, 2014). In addition to this, our research has demonstrated the importance of how the ideological message is conveyed since it can make the emergence of the bias more or less likely.

### Limitations and Future Directions

Although confirming solid recent findings and extending these contributions in an intriguing way, the present results should be taken with caution. The relative small sample size and the specificity of the context in which data were collected, should push researchers to look for further evidence that could be generalized or compared to other contexts. For instance, in countries with a bi-party system (e.g., United States) or with a less polarized political context (e.g., Germany, where left and right-wing parties lean more toward the center) researchers might find different results either with respect to the asymmetry that we found on political bias but also regarding how different stimuli are able to convey a certain ideology. Moreover, our study focused on the Left-Right ideological dichotomy and we did not investigate the single parties because of the high fragmentation of the current Italian political context. Future studies might focus on the political intergroup bias at the single party level. This would be particularly relevant because the last elections showed a fragmented political scenario in which a new – and ideologically not clearly defined-political movement gained a large consensus (e.g., the “Movimento 5 Stelle” became the first party; Ministero dell’Interno, 2018)^2^. Relatedly, the complexity of the current political Italian context might be reflected in different expression of the bias depending on what parties are compared. This might result in different outcomes with respect to what we found by comparing ideologies rather than parties. Future studies should also take into account variables different from those considered in our studies (i.e., perceived threat, entitativity, and agentivity). In facts, the partial mediation resulting from our model suggests that other variables may be at play in the process. Ingroup identification, for example, can moderate responses to threat, leading people who strongly identify with their ingroup to be more sensitive to those threats undermining the distinctiveness or values of the ingroup itself (Voci, 2006). Moreover, people who strongly identify with the ingroup showed greater bias toward the outgroups (Hodson et al., 2003; Jetten et al., 2004; Schmid and Muldoon, 2015). Thus, one further explanation of the difference found in the perceived threat between the two political groups could be that right-wing people might identify less with their ingroup than left-wing people, showing less threat and consequently a lower bias. On the same line, political involvement could be another factor to consider since it might signal ingroup identification which, in turn, might affect the expression of the bias (Hodson et al., 2003; Jetten et al., 2004). Self-esteem, may also play a role, as people with low self-esteem may be more sensitive to threat directed at their own group; as a consequence, they could be more likely to protect the self by expressing ingroup favoritism (possibly also outgroup derogation) as posited by the Social Identity Theory (Tajfel and Turner, 1979).

Another important limitation of the present work that might have prevented us to find results also at the behavioral level is the use of self-report measures. Asking directly to people about their psychological mechanisms is something that have been acknowledged to impair reliability and even the expression of some effects because of their tendency to convey a positive image of themselves (i.e., social desirability) and because of their poor introspective abilities (Nisbett and Wilson, 1977). Future studies might overcome this issue by combining more implicit measures (e.g., IAT Greenwald et al., 1998, or the AMP Payne et al., 2005; Greenwald et al., 2009) and by employing tasks addressing directly the behavior of interest, such as economic investment games, e.g., dictator and trust game or prisoner’s dilemmas (Rapoport and Chammah, 1965; Bolton et al., 1998; Diekmann, 2004; Halevy et al., 2006).

As a final remark, while gender differences may in principle play a relevant role in intergroup relations, studies on this issue does not provide univocal results. Indeed, on the one hand, there is evidence highlighting how men show higher intergroup bias than women (Hewstone et al., 2002), possibly because of men’s higher social dominance tendencies (Pratto et al., 2011); on the other hand, other studies indicate that females express more ingroup bias when facing other females because their balanced gender identity allows them to have a cognitive mechanism that promotes own-group preferences (Rudman and Goodwin, 2004). Moreover, as predicted by ideological conflict hypothesis, when political groups are confronted (like in each of our three studies) intergroup bias should be equally expressed because conflicting values of the outgroup lead to perceive it as threatening (Brandt et al., 2014). In this regard, past research showed that females seem to be more sensitive to social cues and to threatening stimuli (McClure et al., 2004). Thus, we might speculate that left-wing females would perceived the political outgroup as more threatening and, in turn, express higher intergroup bias than their male counterparts. Despite interesting, the present research could not address this relevant issue; first because of its intrinsic characteristics (i.e., for certain stimuli that we used, such as ideological word, ingroup–outgroup own-gender preferences exhibited by females could not be tested because sex-based evaluations are not possible), second because of limitations due to our sample size.

## Data Availability Statement

Complete datasets of the studies can be found at this link: https://data.mendeley.com/datasets/78w49xgk5n/1.

## Author Contributions

MS, GP, SA, and MP conceived and designed research. MS, GP, and MP analyzed the data. MS, GP, IB, SA, and MP interpreted the results. MS prepared the figures. MS, GP, and MP drafted the manuscript. MS, GP, IB, SA, and MP edited and revised the manuscript. MS, GP, IB, SA, and MP approved final version of the manuscript.

## Conflict of Interest Statement

The authors declare that the research was conducted in the absence of any commercial or financial relationships that could be construed as a potential conflict of interest.

## Footnotes

1 https://roma.corriere.it

2 http://elezioni.interno.gov.it/camera/scrutini/20180304/scrutiniCI

## Appendix

### Study 1

#### Right-Wing Politicians:

Angelino Alfano, Mara Carfagna, Nunzia De Girolamo, Maurizio Gasparri, Maria Stella Gelmini, Ignazio La Russa, Roberto Maroni, Giorgia Meloni, Alessandra Mussolini, Stefania Prestigiacomo, Laura Ravetto, Antonio Razzi, Matteo Salvini, Daniela Santanchè, Denis Verdini, Anna Maria Bernini, Laura Comi, Micaela Biancofiore, Beatrice Lorenzin, Clemente Mastella, Silvio Berlusconi, Roberto Formigoni, Maurizio Lupi, Pier Ferdinando Casini, Mario Borghezio, Roberto Calderoli, Renato Brunetta, Paolo Romani, Raffaele Fitto, Umberto Bossi.

#### Left-Wing Politicians:

Nicola Vendola, Pierluigi Bersani, Rosaria Bindi, Laura Boldrini, Maria Elena Boschi, Paola Concia, Giuseppe Civati, Stefano Fassina, Anna Finocchiaro, Marianna Madia, Ignazio Marino, Matteo Renzi, Emma Bonino, Simona Bonafè, Paola Picerno, Alessandra Moretti, Federica Mogherini, Stefania Giannini, Giuliano Poletti, Paolo Gentiloni, Antonio Di Pietro, Dario Franceschini, Graziano Delrio, Walter Veltroni, Romano Prodi, Enrico Letta, Giorgio Napolitano, Sergio Mattarella.

### Study 2

#### Right-Wing Words^∗∗^:

Tradition, Hierarchy, Authority, Competition, Conservatism, Control, Dominance, Order, Conformity, Preservation, Religiosity, Safety, Chastity, Obedience, Patriotism, Nationalism, Certainty, Dogmatism, Convention, Stability, Meritocracy, Protection, Individuality.

#### Left-Wing Words^∗∗^:

Progress, Cooperation, Hospitality, Assistance, Help, Multiculturalism, Peace, Mobility, Equality, Liberalism, Change, Reform, Flexibility, Tolerance, Secularism, Community, Diversity, Solidarity, Freedom, Sharing, Ecology, Redistribution, Innovation.

^∗∗^words were presented in Italian, here translated in English.

### Study 3

**Entitativity items^∗^:** rated on a scale ranging from 1 (*not at all*) to 9 (*extremely*).

(a) “Some groups have more the “group characteristics” than others do. To what extent do left/right-wing people qualify as a ‘group’?”
(b) “To what extent do you think left/right-wing people feel that they are part of their group?”
(c) “How cohesive are left/right-wing people?”
(d) “How organized are left/right-wing people?”
(e) “How much group unity do you think left/right-wing people feel?”
(f) “How much do left/right-wing people interact with one another?”
(g) “To what extent are left/right-wing people interdependent (i.e., dependent on each other) for achieving the group’s goals?”
(h) “How important is the group to left/right-wing people?”

**Agentivity items^∗^:** rated on a scale ranging from 1 (*not at all*) to 9 (*extremely*).

(a) “To what extent are left/right-wing people able to influence other people (i.e., non-Conservatives/Liberals)?”
(b) “To what extent are left/right-wing people able to achieve their goals?”
(c) “To what extent are left/right-wing people able to act collectively?”
(d) “To what extent can left/right-wing people make things happen (e.g., produce outcomes)?”

**Perceived Threat items^∗^:** rated on a scale ranging from 1 (strongly disagree) to 7 (strongly agree).

(a) “If left/right-wing people were to take power, they would work towards the benefit of their group.”
(b) “I feel threatened when left/right-wing people are in power in Italy”
(c) “In certain areas I would be afraid of being identified as left/right-wing people.”
(d) “When I see left/right-wing people symbols in an area, I feel as though my left/right-wing people identity is under threat.”
(e) “I feel threatened when left/right-wing people express their identity and celebrate their cultural traditions.”

^∗^Adaptation from the original items. Items were then translated and presented to participants in Italian.

**Emotional Intergroup Bias^∗∗^:** rated on a 7 points scale with the two words representing the extremes of the scale.

Warmth-Coldness, Negativity-Positivity, Friendliness-Hostility, Suspicion-Trust, Respect-Contempt, Admiration-Disgust.

**Cognitive Intergroup Bias^∗∗^:** rated on a 10 points scale ranging from 1 (not at all) to 10 (very much) (Chambers and Melnyk, 2006).

Intelligent, Trustworthy, Honest, Ignorant, Friendly, Stubborn, Aggressive, Ethical, Considerate, Immoral, Tolerant, Radical.
